# Anaphylaxis Caused by Swimming: A Case Report of Cold-induced Urticaria in the Emergency Department

**DOI:** 10.5811/cpcem.2021.4.51164

**Published:** 2021-07-25

**Authors:** Nicholas M. McManus, Robert J. Zehrung, Trevor C. Armstrong, Ryan P. Offman

**Affiliations:** Mercy Health Hospital, Department of Emergency Medicine, Muskegon, Michigan

**Keywords:** Case report, physical urticaria, cold-induced urticaria, anaphylaxis, angioedema

## Abstract

**Introduction:**

Cold-induced urticaria is a subset of physical urticaria that presents as wheals or angioedema in response to cold exposure. While most cases are idiopathic, secondary associations with infections, medications, and certain cancers have been described.

**Case Report:**

We discuss the case of a 50-year-old male with recent episodes of urticaria from cold air exposure following a flu-like illness six months prior, who presented with symptoms of anaphylaxis upon jumping into a lake.

**Conclusion:**

While the majority of patients develop localized symptoms, understanding this disease entity is imperative as up to one-third of patients can develop severe symptoms including anaphylaxis, particularly from water submersion during activities such as swimming.

## INTRODUCTION

**Figure 1:**
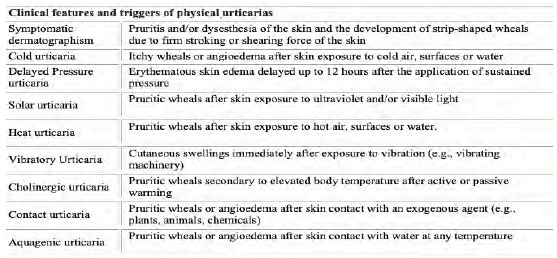
Clinical features and triggers of physical urticarias.^1^,^3^,^4^

Physical urticaria is the term given to a spectrum of conditions where urticaria or angioedema develops in response to a physical stimulus.^1^,^2^ This may occur following exposure to heat (heat-induced urticaria), cold (cold-induced urticaria), water of any temperature (aquagenic urticaria), sunlight (solar urticaria), vibrating machinery (vibratory urticaria), elevated body temperature (cholinergic urticaria), firm stroking or scratching of the skin (dermographism), or in a delayed form up to 12 hours after application of pressure (delayed pressure urticaria).^1^,^3^,^4^ Obtaining a detailed history in regard to exposure to the various triggers seen in physical urticarias is essential to establish a diagnosis. A summary of the various physical urticarias and their triggers are summarized in Figure 1.

The majority of patients with cold-induced urticaria (CIU) develop localized urticarial wheals or cutaneous angioedema within minutes of cold exposure.^5^ However, awareness of this disease process should be of particular interest to emergency physicians as nearly 37% of individuals may experience systemic symptoms ranging from generalized urticaria to anaphylaxis.^4^,^5^,^6^,^7^,^8^,^9^ We report the case of a patient presenting to the emergency department (ED) with symptoms suggestive of anaphylaxis immediately upon jumping into a lake in the middle of summer.

## CASE REPORT

A 50-year-old male presented to the ED with complaints of reoccurring syncope, diffuse urticaria, shortness of breath, vomiting, and reported hypotension by palpation after jumping into a lake. On the day of presentation to the ED, the ambient air temperature was 85° Fahrenheit, while the surface temperature of the lake into which the patient jumped was 68°F. Immediately upon jumping into the lake, bystanders observed the patient to have altered mentation, respiratory distress, and inability to swim back to the boat, although his head remained above water. After being pulled onto the boat, he was described as hyperemic and was noted to be dyspneic. The patient had multiple episodes of syncope along with nausea and vomiting during transport to the ED. He was administered diphenhydramine prior to arrival. Upon arrival to the ED, a diffuse urticarial rash was appreciated. However, further systemic symptoms had improved as he no longer demonstrated respiratory distress, nausea, vomiting, lightheadedness, or syncope. His lungs were clear to auscultation, heart rate was mildly tachycardic at 108 beats per minute and his reported hypotension was improving with a triage blood pressure of 95/67 millimeters of mercury. Epinephrine was deferred due to his rapidly improving symptoms following diphenhydramine administration and removal of the suspected trigger.

Further questioning revealed the patient to have no chronic medical conditions. He did not carry a formal diagnosis of CIU. However, he described symptoms consistent with CIU as he reported that he had several episodes of an unexplained skin eruption recurring on skin exposed to cold air for the prior six months (following a flu-like illness the previous winter). His symptoms would resolve with over-the-counter diphenhydramine, and therefore he never sought medical attention. Further, since the warmer months, he had not had any ongoing episodes until ED presentation.

His ED course was uncomplicated. After complete resolution of his symptoms, the patient was provided education about cold urticaria and told to avoid water submersion. An epinephrine autoinjector was prescribed along with daily antihistamines and he was referred to an allergist for follow-up.

## DISCUSSION

Cold-induced urticaria is the development of wheals or angioedema in response to cold air, liquid, or solid objects.^7^,^10^ According to international guidelines from the 2016 consensus recommendations of the European Academy of Allergology and Clinical Immunology/ the Global Allergy and Asthma European Network/ the European Dermatology Forum/the Urticaria Network e.V., CIU falls under a subset of chronic inducible urticarias.^3^ While considered rare, with a reported prevalence of 0.05%, CIU makes up 5–34% of physical urticaria subtypes, second only to dermographism.^1^,^2^,^7^,^9^

**Educational Merit Capsule**

What do we already know about this clinical entity?

*Most patients with cold-induced urticaria develop localized urticarial wheals or cutaneous angioedema within minutes of cold exposure*.

What makes this presentation of disease reportable?

*Awareness of possible triggers are important as 37% of these patients may experience systemic symptoms, including anaphylaxis after an inconspicuous exposure*.

What is the major learning point?

*While management is not dissimilar from other causes of anaphylaxis, recognition of a physical trigger is imperative as recurrent exposure could be lethal*.

How might this improve emergency medicine practice?

*Obtaining a detailed history in regard to exposure to the various triggers seen in physical urticarias is essential to establish a diagnosis*.

**Figure 2:**
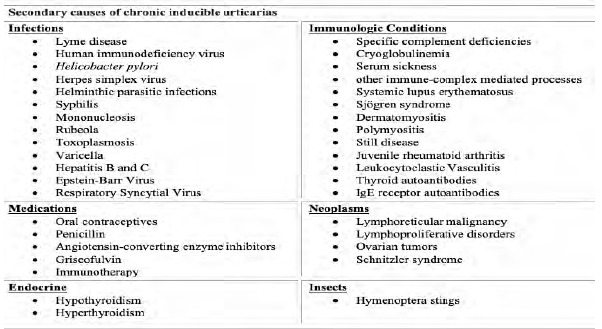
Secondary causes of chronic inducible urticarias.^2^,^7^,^12^,^15^ *Ig*, Immunoglobulin.

The acquired onset of CIU is most common in early adult life with a mean age onset of 22 years. Up to 25% of cases occur in childhood.^1^,^5^,^6^,^11^ Some studies suggest a higher prevalence in females as well as a higher incidence in geographic areas with cooler climates.^2^,^5^,^11^ The majority of CIU cases never have an identifiable cause. Secondary causes have been associated with a wide range of possible etiologies. These include infections (Lyme disease, human immunodeficiency virus, *Helicobacter pylori* colonization, syphilis, mononucleosis, rubeola, toxoplasmosis, varicella, hepatitis); medications (oral contraceptives, penicillin, angiotensin-converting enzyme inhibitors); cryoglobulinemia; Hymenoptera stings; hematologic malignancies; and immunotherapy.^2^,^7^,^12^ A familial variant with autosomal-dominant transmission has also been identified and tends to portray a higher morbidity.^7^,^9^ See Figure 2 for a summary of secondary causes of chronic urticaria.

The exact pathogenesis of CIU has not been well established. Current hypotheses include the presence of immunoglobulin (Ig) E autoantibodies that react against specific skin antigens only at low temperatures, leading to activation and degranulation of skin mast cells and the resultant release of histamine and other proinflammatory mediators.^1^,^6^,^7^,^10^ While the long-term prognosis of acquired forms of CIU is generally good, the condition can lead to a diminished quality of life for a large number of patients. Some studies suggest that a self-limited remission can be achieved in up to 50% of patients with acquired disease within 5–6 years, while others are less positive reporting that only 25% have resolution of symptoms by 10 years.^2^,^7^,^9^,^11^ In patients who achieve remission, recurrence is unlikely; however, the majority of patients with familial variants will have symptoms lasting a lifetime.^7^,^9^

Most patients develop localized urticarial wheals or cutaneous angioedema within minutes of exposure to cold air, surfaces, or water.^5^ However, nearly 37% of patients may experience systemic symptoms including generalized urticaria, headache, fatigue, respiratory distress, or anaphylaxis with cardiovascular compromise, most commonly following swimming.^4^,^5^,^6^,^7^,^8^,^9^ A distinct subtype of CIU (delayed type) in which patients may not develop symptoms for 24–72 hours after cold exposure also deserves recognition in the emergency medicine literature.^7^,^12^

For those attempting to establish a diagnosis in the ED, guidelines from the 2016 international consensus recommendations suggest appropriate diagnostic testing be done through cold provocation testing using either the ice cube test or measurements using TempTest (Professor Marcus Maurer, Charité – Universitätsmedizin Berlin, Berlin,Germany).^1^,^3^ With the ice cube test, the clinician places an ice cube on the forearm for 3–5 minutes and then it is removed for 10 minutes. The clinician then observes for development of wheals after rewarming, indicating a positive test with sensitivity of 83–90% and a specificity of 100%.^3^,^7^,^10^ Alternatively, cold provocation can be done with a device such as the TempTest, where the patient is exposed to a variety of temperatures yielding similar diagnostic accuracy to the ice cube test (sensitivity of 93% and specificity of 100%).^7^ While unlikely to be available in the ED, devices like the TempTest enable clinicians to both establish a diagnosis and determine the threshold temperature at which symptoms occurs for a given patient.^7^,^10^,^13^

Cold provocation testing using ice packs or cold-water baths is not recommended due to the risk of systemic response with the increased percentage of surface area affected.^12^,^13^ It is worth mentioning that a subset of patients will have a negative cold provocation test, which does not exclude the diagnosis, as atypical variants are possible.

Acute treatment of patients presenting to the ED is with antihistamines and, if symptoms are suggestive of anaphylaxis, epinephrine.^2^ Intravenous fluids should be warmed, as some patients’ thresholds for histamine release may be above that of room temperature fluids.^8^ Patients should be counseled on avoiding over-exposure in cold weather and to avoid cold water submersion.^7^,^14^,^15^ If patients do not know their personal temperature threshold, counseling can include avoiding water temperatures less than 77°F (25°C), as temperatures above this threshold are generally considered safe for most patients.^8^ Further, patients should be advised that cold food and beverages should be avoided as they could induce oropharyngeal angioedema or anaphylaxis.^11^ Patients who present with systemic or atypical symptoms should be prescribed an epinephrine autoinjector and referred to an allergy/immunology specialist for diagnostic testing and chronic management.^7^,^14^

A short (less than 10 days) course of corticosteroids may be considered for other types of chronic urticaria; however, the literature has not shown this to be beneficial in preventing the histamine release seen in the cold-induced urticaria subset of these patients and is therefore not routinely recommended.^7^,^15^ Addition of a histamine receptor (H2)-antihistamine blocker, such as famotidine or cimetidine, may be helpful in patients refractory to H1-antihistamine monotherapy.^15^,^16^

**Figure 3:**
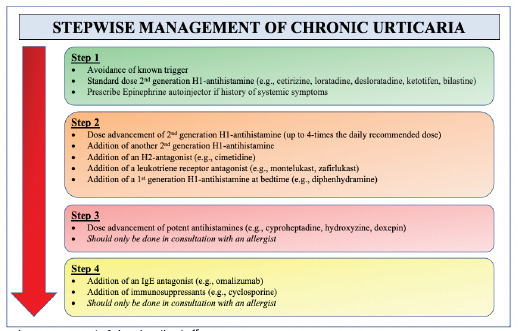
Stepwise management of chronic urticaria.^15^ *H1*, histamine-1 receptor, *H2*, histamine-2 receptor.

Beyond the acute management of CIU, chronic treatment is largely aimed at avoidance of cold and prevention of symptoms with prophylactic dosing of second-generation H1-antihistamine blockers (eg, cetirizine, loratadine, desloratadine, ketotifen, bilastine).^2^,^3^,^10^,^12^,^15^ In 2014, a joint task force representing the American Academy of Allergy, Asthma & Immunology; the American College of Allergy, Asthma & Immunology; and the Joint Council of Allergy, Asthma & Immunology published a practice parameter update describing a stepwise approach to the general management of chronic urticaria^15^ (Figure 3). H1-antihistamine dosing should begin at standard dosing and titrated upward as needed. High dose regimens (up to four times the daily recommended dose) are often needed; studies have not shown an increase in adverse events with this regimen.^10^,^15^ For patients with symptoms refractory to high-dose antihistamines, the addition of another second-generation antihistamine, an H2-antagonist (ie, cimetidine), a leukotriene receptor antagonist (ie, montelukast, zafirlukast) or the addition of a first-generation antihistamine (ie, diphenhydramine) taken at bedtime can be considered. For patients refractory to these measures, dose advancements of potent antihistamines (ie, cyproheptadine, hydroxyzine or doxepin) could be considered followed by the addition of an IgE antagonist (omalizumab) or immunosuppressant (ie, cyclosporine), but should only be started in conjunction with an allergy clinician.^3^,^7^,^10^,^12^,^15^ While systemic corticosteroids are frequently used, no controlled studies have shown efficacy for chronic urticarias, and their long-term use is not recommended.^15^

## CONCLUSION

Cold-induced urticaria is a rare disease process with the potential to carry significant morbidity and even mortality if it is not properly identified upon initial presentation to the ED. When patients present to the ED with concerns for allergic reaction, it is important for the clinician to assess for the presence of physical triggers when obtaining a detailed history, particularly when the underlying etiology is not apparent. While the emergent management of CIU is similar to other causes of allergic response or anaphylaxis, recognition of cold exposure as an etiology is imperative as recurrent exposure, particularly during swimming, could be lethal.
